# Intraoperative radiotherapy in gynaecological and genito-urinary malignancies: focus on endometrial, cervical, renal, bladder and prostate cancers

**DOI:** 10.1186/s13014-016-0748-x

**Published:** 2017-01-19

**Authors:** Marco Krengli, Carla Pisani, Letizia Deantonio, Daniela Surico, Alessandro Volpe, Nicola Surico, Carlo Terrone

**Affiliations:** 1 Department of Translational Medicine, University of Piemonte Orientale, Via Solaroli, 17-28100 Novara, Italy; 2 Department of Radiotherapy, University Hospital Maggiore della Carità, Novara, Italy; 3 Department of Obstetrics and Gynecology, University Hospital Maggiore della Carità, Novara, Italy; 4 Department of Urology, University Hospital Maggiore della Carità, Novara, Italy

**Keywords:** Intraoperative radiotherapy, Endometrial cancer, Cervical cancer, Renal cancer, Bladder cancer, Prostate cancer

## Abstract

Intraoperative radiotherapy (IORT) refers to the delivery of a single radiation dose to a limited volume of tissue during a surgical procedure. A literature review was performed to analyze the role of IORT in gynaecological ﻿and genito-urinary cancer including endometrial, cervical, renal, bladder and prostate cancers.

Literature search was performed by Pubmed and Scopus, using the words “intraoperative radiotherapy/IORT”, “gynaecological cancer”, “uterine/endometrial cancer”, “cervical/cervix cancer”, “renal/kidney cancer”, “bladder cancer” and “prostate cancer”. Forty-seven articles were selected from the search databases, analyzed and briefly described.

Literature data show that IORT has been used to optimize local control rate in genito-urinary tumours mainly in retrospective studies. The results suggest that IORT could be advantageous in the setting of locally advanced and recurrent disease although further prospective trials are needed to confirm this findings.

## Background

Intraoperative radiotherapy (IORT) refers to the delivery of a single large dose of radiation to a limited volume of tissue during a surgical procedure.

Radiotherapy (RT) has a major role in the management of most gynaecological and genito-urinary cancer as adjuvant or neoadjuvant treatment or as radical treatment in combination with chemotherapy or hormone therapy. IORT has the capability to increase the radiation dose with very limited or no increase of toxicity thanks to the target exposition during the surgical procedure. For this reason, IORT can be used in various settings of gynaecological and genito-urinary tumours aiming at dose intensification and consequently at increasing tumour control rate.

IORT can be delivered using dedicated linear accelerator producing electron beams, X-rays sources delivering low-energy radiation or high dose-rate brachytherapy units through catheters positioned in the tumour bed and loaded with iridium-192. In particular, electrons generated by linacs and brachytherapy sources can be conveniently used for IORT procedures in gynaecological and genito-urinary tumours.

Interestingly, the first IORT experience was indeed reported in 1905 for the treatment of a 33 year old woman affected by uterine carcinoma [1]. Over the following decades, IORT was increasingly used for several tumours including gynaecological and genitor-urinary malignancies.

In 1998, the International Society of Intraoperative Radiation Therapy (ISIORT) was founded in order to promote a scientific and professional approach to IORT activity. Among their other activities, ISIORT-Europe collected and recorded information regarding IORT treatments, including those of gynaecological and genito-urinary cancers, from the affiliated centres in a database registry [2, 3].

This review focuses on the use of IORT in genito-urinary malignancies, reporting tumour setting and outcome for endometrial, cervical, renal, bladder and prostate cancers.

## Research criteria

Literature search was performed through Pubmed and Scopus databases by using the following key words: “intraoperative radiotherapy/IORT”, “gynaecological cancer”, “uterine/endometrial cancer”, “cervical/cervix cancer”, “renal/kidney cancer”, “bladder cancer” and “prostate cancer”. Eighty-four articles were found from 1981 to 2015. Reviews and case reports were excluded as well as clinical series presented as abstract at conferences proceedings. Forty-seven articles were finally selected for the review.

## Endometrial and cervical cancers

Patients with endometrial and cervical cancer are usually treated with surgery and RT with or without chemotherapy depending on risk factors. After primary treatment, the risk of local failure is up to 60% [4] and the options for a new treatment are surgery, RT when a reirradiation is feasible, and chemotherapy. After such treatments, disease control has been reported in 25–50% and 18–47% in patients with recurrent endometrial and cervical cancer, respectively [5]. In these recurrent patients, IORT after surgical resection can been considered to increase the probability of local control, especially when a repeated course of EBRT is not feasible. This treatment approach including IORT is reported in the NCCN guidelines with an evidence of category 3 [6].

The use of IORT in the management of endometrial and cervical cancer was explored in 15 studies, most of them analysing retrospectively patients affected by locally advanced primary and recurrent disease. The majority of articles reported on the clinical experience from the Mayo Clinic and the University Hospital Gregorio Marañón in Madrid [7–21] (Table 1). In these clinical series, IORT was delivered to the tumour bed with electrons in the majority of cases and with low kV x-rays or brachytherapy through catheters implanted during the surgical procedure and uploaded with iridium wires in postoperative setting in selected patient series.

**Table 1:** IORT studies for endometrial and cervical cancer

Reference	N.pts	Type of cancer	Primary/recurrent	EBRT N. pts Dose (Gy)	IORT dose (Gy)	Technique	Median follow-up months(range)	Local Control	Overall Survival	Toxicity
Sole [7]	61	Uterus 18 Cervix 32 Other 11	Pelvic recurrent 35 (57%) Paraortic recurrent 26 (43%)	Mean 31 Gy (29–45)	R0: 10–12.5 Gy R1: 15 Gy	IOERT	42 (2–169)	5-years 65%	5-years 42%	RTOG acute ≥ G3: 23 RTOG late ≥ G3: GI 8 GU 3 Neuropathy 1
Foley [8]	32	Cervix 21 Uterus 6 Other 5	Pelvic recurrent 26 (81%) Primary 6 (19%)	NA	Mean 13.5 Gy (10–22.5)	IOERT	Median 26 (3–196)	5-years R1 73% 5 years R2 71%	5-years 70% R1 77% R2 55%	≥G3 47% 5 IORT-related GU 2 Bone 1 Lymphedema 2
Backes [9]	32 21 IORT	Cervix 21 Other 11	Recurrent 32 (100%)	6 pts, mean 26 Gy (10–40)	Median 17.5 Gy (10–20 Gy)	IOERT HDR IORT	NA	Median PE + IORT 10 months LEER + IORT 9 months PE 33 months	Median PE + IORT 10 months LEER + IORT 17 months PE 41 months	NA
Barney [10]	86	Cervix	Pelvic recurrent 73 (85%) Primary 13 (15%)	61 pts (71%) No prior RT: median 45 Gy Prior RT: median 39.6 Gy	median 15 Gy (6–25 Gy)	IOERT	32 (1–306)	3-years 62%: 70% primary 61% recurrent	3-years 25%	≥G3 GI 4 GU 1 Neuropathy 1 Other 4
Calvo [11]	35	Uterus 7 Cervix 20 Other 8	Pelvic recurrent 35 (100%)	16 pts: 45 Gy no previous RT 30.6 Gy previous RT	R0: 10–12.5 Gy R1: 15 Gy	IOERT	46 (3–169)	5-years 58%	5-years 42%	acute ≥3: 14 late ≥3: GI 5 GU 2 Neuropathy 1
Giorda [12]	35	Cervix	Primary 35 (100%)	neoadj 50.4 Gy	Mean 11 Gy (10–15)	IOERT	NA	2-years 89%	5-years 49%	Peri/post-surgery GU 10
Tran [13]	36	Cervix 17 Uterus 11 Other 8	Recurrent 32 (88%)	18 pts (50%) mean 44 Gy	Median 11.5 Gy (6–17.5)	Orthovoltage-IORT	Mean 50 (2–198)	5-years 44% Cervix 45% Uterus 58%	5-years 42%	≥G3 10 pts 28%
Dowdy [14]	25	Uterus	Recurrent 25 (100%)	21 pts 45 Gy	Median 15 Gy (10–25 Gy)	IOERT	Median 34	84%	5-years: 71% R0 47% R1 0% R2	Neuropathy 8 GU 5 Fistulas 5 Bone fractures 2
Awtrey [15]	27	Uterus	Pelvic Recurrent 27 (100%)	12 pts	NA	IOERT 9 pts	Median 24 (5–84)	NA	2-years 78%	NA
Martinez-Monge [16]	67	Cervix	Pelvic Recurrent 36 (54%) Primary 31 (46%)	36 pts : 45 Gy	Primary: 12 Gy median (10–25) Recurrent: 15 Gy (10–20)	IOERT	Primary: 58 (8–144) Recurrent 19 (1–138)	10-year 69%: 93% primary 47% recurrent	10-year 35%; 58% primary 14% recurrent	15% IORT related
Gemignani [17]	17	Cervix 9 Uterus 7 Other 1	Recurrent 17 (100%)	2 pts dose NA	Mean 14Gy (12-15Gy)	HDR-IORT	20 (3–65)	67	54	NA
DelCarmen [18]	15	Cervix 5 Uterus 3 Other 7	Pelvic Recurrent 14 (93%) Primary 1 (7%)	-	10-22.5 Gy	IOERT	(3–36)	54%	74%	Neuropathy 4 GU 3 Lymphedema 2
Garton [19]	39	Cervix 22 Uterus 10 Other 7	Pelvic Recurrent 36 (92%) Primary 3 (8%)	28 pts Median 45 Gy (1–67)	Median 17.3 Gy (10–25 Gy)	IOERT	Median 25 (6–125)	5-years 67%	5-years 32%	≥G3 14 (36%) IORT related 6
Mahè [20]	70	Cervix	Pelvic Recurrent 70 (100%)	30 pts (20–45)	R0 mean 18 (10–25) R1-biopsy mean 19 (10–30)	IOERT	Mean 15 (2–69)	21% R0 27% R1-2 11%	3-years 8%	10-IORT related GI 1 GU 4 Neuropathy 5
Stelzer [21]	22	Cervix	Pelvic Recurrent 22 (100%)	6 pts: 26–50 Gy 7 pts: 45–62.4 Gy	22 Gy median (14–27.8 Gy)	IOERT	Minimum 15 months	5-years 48%	5-years 43%	Neuropathy 7

*Pts* patients, *IORT* Intraoperative radiotherapy, *IOERT* intraoperative electron radiotherapy, *EBRT* external beam radiotherapy, *GU* genitourinary *GI* gastrointestinal, *NA* not available, *R0* negative margins, *R1* microscopic residual disease, *R2* macroscopic residual disease

In endometrial cancer patients, limited loco-regional recurrences have a relatively high control rate of about 60% at 5 years either with pelvic exenteration or local EBRT in non-previously irradiated patients [22, 23]. In this tumour setting, the use of IORT was reported in retrospective studies [14, 15]. Dowdy et al. [14] found that radical resection of the pelvic sidewall with negative margins and IORT resulted in a relatively high overall survival rate (71%) (Table 1). Awtrey et al. [15] reported that the addition of IORT to cytoreductive surgery in 27 recurrent endometrial cancer patients resulted in a 2-year disease free survival (DFS) rate of 78% versus 67% when IORT was not used, although this difference was not statistically significant. Based on these retrospective data, the addition of IORT to surgery could be proposed in patients with isolated endometrial cancer recurrences, especially when margins might be close or microscopically positive.

Patients with a loco-regional recurrence of cervical cancer and candidates for salvage surgery can undergo also IORT with the intent to sterilize the possible residual disease and improve the outcome. This approach was described in three series from Mahe et al. [20], Barney et al. [10] and Martinez-Monge et al. [16] who reported globally the results in 188 patients with recurrent cervical cancer. Intraoperative radiation dose ranged from 6 Gy to 30 Gy, with higher doses in case of macroscopically positive margins (R2). Mahe et al. [20] reported a slightly higher local control, although statistically not-significant, in patients with radical resection versus those who received partial resection (27% vs. 11%), Barney et al. [10] did not observe any influence of margins status for local control and Martinez-Monge et al. [16] reported a risk of distant metastases of 38% in patients with negative margins (R0) and 100% in those with macroscopic residual disease (R2). From these studies, it emerged that the status of the margins is the most important risk factor for treatment and the association of IORT seems to improve the probability of local control.

As far as locally advanced primary cervical cancer is concerned, two series treated by IORT are reported in the recent literature [12, 16]. In both studies, patients underwent radical hysterectomy and 10–25 Gy IORT after neoadjuvant EBRT, concomitantly to chemotherapy, to a total dose of 50.4 Gy. In the Giorda's phase II trial, patients tolerated radio-chemotherapy quite well, but developed high incidence of toxicity (79%) after surgery and IORT [12]. In the Martinez-Monge's retrospective series, 15% of side effects were related to IORT [16]. The available data suggests that this aggressive strategy is not advantageous in particular for the risk of severe side effects and that concomitant radio-chemotherapy alone should be considered the best treatment strategy in this patient setting [6].

In conclusion, literature data supports the use of IORT in recurrent endometrial and cervical cancer to improve local control whereas its use appears more controversial in primary locally advanced disease. The potential benefit of this approach is mainly based on retrospective mono-institutional studies and should be further verified by prospective possibly randomized trials investigating the potential advantage compared to EBRT alone.

## Renal cancer

Historically, the standard therapy for renal cell carcinoma is radical nephrectomy. Local control and survival rates after surgery alone are satisfactory for T1-T2 N0 with rates of 90-100% and 80-90% at 5 years, respectively. The results are less favourable for locally advanced and N+ disease, where the 5-year local control rate and overall survival rates are 70-80% and 0-40%, respectively. In renal cancer, the isolated local recurrence after radical nephrectomy is uncommon (0.7-3.6%) but it is associated with a poor prognosis. An aggressive surgical approach to local advanced or recurrent disease, possibly including the removal of the renal fascia and leading to negative margins, seems to improve outcome and prolong survival [24, 25].

Although renal cell carcinoma has traditionally been considered relatively radiation resistant, recent data using hypofractionation for primary or metastatic lesions suggest that this resistance can be overcome by high dose per fraction, as used in the IORT scenario [26].

The role of IORT in the management of renal cancer was explored in a number of retrospective studies with patients presenting with locally advanced primary or recurrent disease [27–33] (Table 2). IORT doses varied from 10 to 25 Gy depending on the amount of residual tumour after maximal resection and on the dose of the combined EBRT. All cases of these series were characterized by postoperative microscopic or macroscopic residual disease in the renal fossa. A more recent study [27] considered 98 patients with advanced or recurrent renal cell carcinoma treated with IORT at nine institutions. Preoperative or postoperative EBRT to a total dose of 40–50.5 Gy was administered to 27% or 35% of patients, respectively. The median radiation dose administered with IORT was 15 Gy (range: 9.5-20 Gy). Overall survival and disease free survival rates at 5 years were quite similar and only 24% of relapses were local whereas 76% were distant. This fact suggests the potential benefit in local control when IORT is added. Similar results in terms of local control rates were reported in previous studies from other institutions (Table 2). In these series, the acute and late toxicity profile seems acceptable. Many studies, however, are characterized by a limited description of late side effects.

**Table 2:** IORT studies for renal cancer

Reference	N. pts	Type of cancer	Primary/recurrent	EBRT	IORT dose (Gy)	Technique	Median follow-up	Local control	Overall survival	Toxicity
Paly [27]	98	Advanced or recurrent renal cell carcinoma	Pelvic locally recurrent 100%	26 pts: 45–40 Gy pre or post surgery	Median dose: 15 Gy (9.5-20 Gy)	IORT	3.5-years (3–169)	5-years 39% advanced disease 5-years 52% recurrent disease	5-years 37% advanced disease 5-years 55% recurrent disease	NA
Habl [28]	17	Locally recurrent disease	Pelvic locally recurrent 100%	-	Median dose: 15 Gy (10–20 Gy)	IORT	18 months	2 years 91%	2 years 73%	No late toxicities
Calvo [29]	25	Advanced or recurrent renal cell carcinoma	Pelvic locally recurrent 100%	15 pts: 44 Gy perioperative	Median dose: 14 Gy (9–15 Gy)	IORT	22.2 years (3.6-26)	5-years 80%	5-years 38% 10-year 18%	6 pts acute/late toxicities ≥ 3
Hallemeir [30]	22	Advanced or recurrent renal cell carcinoma	-	21 pts: 41.5 Gy perioperative	Median dose: 12.5 Gy (10–20 Gy)	IORT	9.9 years (3.6-20)	NA	5-years 40%	5 ptsacute/late toxicities ≥ 3
Master [31]	14	Local recurrent renal cell carcinoma	Pelvic locally recurrent 100%	-	Median dose: 15 Gy (12–20 Gy)	IORT	NA	NA	5 years 30%	NA
Eble [32]	14	Advanced or recurrent renal cell carcinoma	-	14 pts:40 Gy postoperative	15-20 Gy	IORT	24.3 months	NA	11.5 months	0%
Frydenberg [33]	11	Local persistence or local recurrent		11 pts: 45–50.4 Gy preoperative	10-25 Gy	IORT	NA	NA	NA	NA

*Pts* patients, *IORT* Intraoperative radiotherapy, *IOERT* intraoperative electron radiotherapy, EBRT external beam radiotherapy, *GU* genitourinary, *GI* gastrointestinal, *NA* not available

From all published data, although from retrospective series, it emerges that the addition of IORT to surgery and EBRT is associated with high rates of local control with acceptable toxicity. The best candidates could be untreated patients with large tumour volume and high risk of positive margins after radical nephrectomy and patients with locally recurrent tumours. The long-term prognosis is mainly related to the risk of onset of distant relapse that is quite common, especially in patients with recurrent disease. This fact advocates the need for additional systemic effective therapy.

## Bladder cancer

The goals of treatment for invasive bladder cancer are high long-term overall and disease-free survival rates with acceptable functional outcome, however, radical cystectomy, that is nowadays the standard, needs urinary diversion and results in erectile impotence and infertility. In order to avoid these adverse effects and preserve quality of life, bladder-preserving treatments have been proposed as a viable option in selected patients [34]. Bladder preservation strategies for muscle invasive bladder cancer evolved over time from single modality to multimodality treatment approaches, including transurethral resection and chemo-radiation protocols. The use of an intraoperative radiation boost by brachytherapy or electrons may be advantageous for intensifying the dose and obtaining local control without compromising organ function.

From the literature databases, 15 studies using IORT by brachytherapy implants or electrons were selected for this review [35–49] (Table 3). Brachytherapy was the most used intra-operative modality and was employed either as a single treatment or as a boost dose combined with EBRT. It may represent a curative treatment for selected high-risk superficial and solitary muscle infiltrating tumours. Clinical target volume (CTV) typically includes the macroscopic disease or the tumour bed with safety margin to full thickness of the bladder wall.

**Table 3:** IORT studiesfor bladder cancer

Reference	N. pts	Stage	EBRT	Treatment	Local control (5 years)	Overall survival 5-years	Toxicity
Hallemeier [35]	11	Local recurrence	Neoadjuvant	Surgery + IORT (12.5 Gy)	51%	16%	NA
Koning [36]	1040	T1-T2	Neoadjuvant	surgery, Ir-192 (25–40 Gy)	75%	62%	Fistula 24, ulcers/necroses 144
van Onna [37]	111	T1-T2	Neoadjuvant	Ir-192 (40 Gy)	NA	70%	Fistula 5 GU 5
van der Steen-Banasik [38]	76	T1-T2	Neoadjuvant	Cs-137, Ir-192 (30–60 Gy)	70%	57%	NA
Blank [39]	122	T1-T2-T3	Neoadjuvant	Ir-192 (20–70 Gy)	76%	73%	GU 5
Nieuwenhuijzen [40]	108	T1-T2	Neoadjuvant	Ir-192	73%	62%	NA
De Crevoisier [41]	58	T1-T2-T3	Neoadjuvant	surgery, Ir-192 (60 Gy)	65%	60%	5 major late toxicities
Gerard [42]	27	T2, T3	No	Surgery + IORT	85%	53%	NA
Pernot [43]	82	T1, T2, T3, T4, Tx	Neoadjuvant	surgery Ir-192 (30–50 Gy	78%	73%	7 late toxcities ≥ G3
Calvo [44]	40	T2, T3, T4	Neoadjuvant	surgery + IORT (15 Gy)	NA	68%	NA
Rozan [45]	205	T1-T2-T3	Neoadjuvant	surgery Ir-192 (30–50 Gy	NA	77.4% T1, 62.9% T2, 46.8% T3	haematuria, fistula, chronic cystitis 29
Batterman [46]	85	T2	Neoadjuvant	Ra-226	74%	55%	NA
Mazeron [47]	24	T2	Adjuvant	surgery, Ir-192	92%	58%	NA
van der Werf-Messing [48]	328	T2	Neoadjuvant	Ra-226	77%	56%	NA
Matsumoto [49]	28	T2	Adjuvant	IORT	82%	62%	NA

*Pts* patients, *EBRT* External beam radiation therapy, *Ra-226* brachytherapy, radium needles, *Ir-192* brachytherapy, afterloading iridium, *IORT* intraoperative electron radiation therapy

All the studies about brachytherapy were retrospective analyses of single or multiple co-operative centres. In 2012, a multicentre survey [36], assessed the role of brachytherapy in 1040 patients with early stage bladder carcinoma in a muldisciplinary setting. Patients were treated by pre-operative EBRT and limited surgery with brachytherapy implant. From this analysis, it emerged that this approach can offer adequate results in terms of local control and overall survival in selected patients suitable (Table 3). In this regard, a careful patient selection is particularly important in relation to the non-negligible probability of acute toxicity leading to fistulas or necrosis.

A recent systematic review with meta-regression analysis showed better results after brachytherapy than after cystectomy in terms of overall survival, but not in terms of cause-specific survival in patients with muscle-invasive bladder cancer. The authors commented that this discrepancy can be explained at least in part by the differences in tumour stage between the two groups [50].

The integration of an IORT boost to the whole bladder in a multidisciplinary protocol combining neoadjuvant systemic chemotherapy, preoperative RT, and planned cystectomy has proven to be feasible in the Pamplona's series [44]. The mean sterilization rate of invasive bladder cancer, confirmed in pathologic studies by the cystectomy specimen, was 65%, and seemed to be increased by the addition of neoadjuvant chemotherapy. This finding can be of importance with respect to the development of new protocols aiming at bladder preservation. In the Lyon series [42], an excellent bladder preservation rate of 69% was achieved with the combination of preoperative chemo-RT followed by IORT. This is the only prospective study about IORT in bladder carcinoma. It could be of interest to attempt verifying these results in further studies using an IORT approach.

In conclusion, after a careful patients selection, IORT could be used within a bladder sparing multidisciplinary approach because of the favourable 5-year local control rates aiming at escalating the radiation dose. IORT might have a role also in case of radical surgery for locally advanced disease in order to improve local control rates, as performed in the Pamplona’s series. Multicentric prospective studies could useful to confirm the role of IORT﻿ in this tumour setting.

## Prostate cancer

The rationale for dose escalation with IORT in prostate cancer is based on the demonstration of a dose–response relationship and a low α/β value in the radiobiological linear quadratic model [51]. Likewise, the exploitation of this principle is being increasingly investigated in EBRT with hypofractionation [52].

Among 14 IORT literature studies, 9 clinical series and the ISIORT registry were selected and presented in Table 4 [2, 53–61].

**Table 4:** IORT studies for prostate cancer

Reference	N. pts	Patients’ selection	Surgical approach	IORT dose (Gy)	Technique	Adjuvant EBRT	BRFS	Overall survival	Toxicity
Krengli (ISIORT) [2]	108	Intermediate-high risk^a^	NA	8-15 Gy with EBRT 18–21 Gy single shoot	IORT or 50-KV	NA	NA	NA	NA
Krengli [53]	38	Intermediate-high risk^a^	Retropubic approach IORT + Prostatectomy	10-12 Gy	IORT	46-50 Gy, 2 Gy/fx	82%	2-years 100%	Lymphocele 16% hematoma 6%
Rocco [54]	33	Intermediate-high risk^a^	Retropubic approach IORT + Prostatectomy	12 Gy	IORT	45 Gy, 1.8 Gy/fx	97%	2-years 100%	GU: 17% ≥ G2 GI: 10% ≥ G2
Saracino [55]	34	Intermediate risk^a^	Retropubic approach Prostatectomy + IORT	16-22 Gy	IORT	No	77%	NA	No GU/GI toxicities ≥ G1
Orecchia [56]	11	High-risk^a^	Retropubic approach IORT + Prostatectomy	12 Gy	IORT	45 Gy, 1.8 Gy/fx	NA	NA	No GU/GI toxicities ≥ G1
Kato [57]	54	Stage B2-D1^b^	Perineal/retropubic No prostatectomy	25-30 Gy	IORT	30 Gy, 2 Gy/fx	74%	NA	Early GI G3: 7%
Higashi [58]	35	Stage B-C ^b^	Perineal/retropubic No prostatectomy	25-30 Gy	IORT	30 Gy, 2 Gy/fx	NA	5-years 87% (stage C) 5-years 92% (stage B)	NA
Abe [59]	21	Stage B2-days ^b^	Perineal	28-35 Gy or 20–25 Gy (if combined with EBRT)	IORT	50 Gy	NA	5-years 72%	GU: 100% early ematuria 10% early pollakiuria
Kojima [60]	30	Stage B-C ^b^	Perineal/retropubic No prostatectomy	--	IORT	NA	NA	5-years 43%	NA
Takahashi [61]	14	Stage B2-days ^b^	Perineal No prostatectomy	28-35 Gy or 20–25 Gy (if combined with EBRT)	IORT	50 Gy	NA	NA	0%

*pts* patients, *GU* genito-urinary, *GI* gastro-intestinal, *BRFS* biochemical relapse-free survival, *NA* not available

^a^National Comprehensive Cancer Network (NCCN) guidelines NCCN [6]

^b^Whitemore-Jewett staging system [Whitmore 1956, Jewett 1975]

Early data on IORT in prostate cancer came from the Kyoto University and the Saitama Cancer Centre in Japan, where the authors treated patients through a perineal IORT approach without prostatectomy [59, 61]. More recent experiences were reported by Italian authors using IORT in combination with radical prostatectomy and regional lymph node dissection before or after the surgical procedure [53–56]. A relevant percentage (81%) of patients was included in prospective institutional study protocols as described in the ISIORT data-registry [2]. From this analysis, it emerged that IORT was used as a boost dose prior to prostate removal in most cases. When a single-shot radiation strategy was adopted, a dose of 18–21 Gy was delivered, similarly to the breast cancer model. The diameter and bevel end angle of the applicators were selected based on target dimensions, considering a margin of at least 5 mm around the prostate and the necessity to reach the target underneath the pubic arch while sparing the bladder. The electron beam energy, between 9 and 12 MeV, depended on the depth of the target and the position of the rectum, which should be spared.

Patient selection varied widely in the various studies. The Japanese series included either early or advanced stage disease and in particular the Kyoto University included stages from A2 to C treated with curative intent and even stage D2 treated with palliative intent [59, 61]. The Italian studies accrued only non-metastatic locally advanced disease based on the identification of preoperative risk factors.

In terms of post-surgical early and late side effects, IORT for prostate cancer resulted an acceptable procedure. In the Japanese series, toxicity resulted in early haematuria, pollakiuria but only very few cases of late chronic cystitis and urethral stricture. Interestingly, Kato et al. reported a reduction in rectal toxicity by using a spacer to reduce the dose to the anterior rectal wall [57].

In the Italian series, surgical complications, such as haematoma and lymphocele, occurred with a similar incidence to that of conventional prostatectomy [53–56]. No major surgical complications were described and patients had no significant difference of estimated blood loss and need of transfusion. In this regard, Rocco et al. reported post-surgical complications in 42% of patients after surgery and IORT and in 30% after prostatectomy alone [54].

Although the relatively short follow-up, the outcome in terms of biochemical disease free survival was quite promising resulting higher than 70% in both the Japanese and Italian series (Table 4). Of note, a recent update of our clinical series of 95 patients showed a 5-years biochemical disease-free survival rate of 78% in high-risk patients (oral presentation at ISIORT-ESTRO Forum, Barcelona, 24–28 April, 2015).

Clinical trials with long follow-up are needed to assess the real efficacy of IORT in locally advanced prostate cancer but preliminary results look quite promising. The best candidates for IORT possibly combined with EBRT, could be the patients staged T3N0 with high risk for positive margins. In the future, multicentre studies should be designed to better clarify the real role of IORT for dose escalation in local advanced prostate cancer patients.

## Conclusions

The delivery of a high single dose of radiation to a limited volume during the surgical time, achievable with IORT, is useful to avoid normal tissues not at risk of microscopic disease. For gynaecological and genito-urinary cancers, IORT is not a standard treatment but it may be considered a treatment option in selected patients.

In endometrial, cervical and renal cancers, IORT can be used mainly in recurrent disease, whereas in bladder carcinoma it may be part of an organ-sparing treatment approach aiming at patient quality of life preservation. In the case of prostate cancer, IORT can be used in locally advanced high risk disease possibly combined with EBRT to intensify the radiation dose in the attempt to improve long term local control and possibly increase biochemical disease-free and overall survival.

The available literature data are interesting but the present review shows that the majority of published clinical studies are mono-institutional, retrospective and often included a limited number of patients. In order to overcome these limitations, large multicentre collaborations should be established to design prospective clinical trials aiming at better defining the role of IORT in tailored multimodality therapeutic approaches for gynaecological and genito-urinary tumours. For this purpose, the ISIORT could serve as a basis for future collaboration and the ISIORT-Registry could be a platform for sharing data and promote clinical research.

## Abbreviations

CTV: Clinical target volume
EBRT: External beam radiotherapy
IOHDR: Intra Operative high dose rate
IORT: Intraoperative radiotherapy
ISIORT: International society of intraoperative radiation therapy
RT: Radiotherapy
